# The association between haemosporidian infection and non-breeding moult location in great reed warblers revisited by combining feather stable isotope profiles and geolocator data

**DOI:** 10.1007/s00442-023-05491-x

**Published:** 2023-12-23

**Authors:** Petr Procházka, Tamara Emmenegger, Silke Bauer, Arif Ciloglu, Dimitar Dimitrov, Bengt Hansson, Dennis Hasselquist, Elizabeth Yohannes, Pavel Zehtindjiev, Staffan Bensch

**Affiliations:** 1 Institute of Vertebrate Biology, Czech Academy of Sciences, Květná 8, 603 65 Brno, Czech Republic; 2 Molecular Ecology and Evolution Lab, Department of Biology, Lund University, Sölvegatan 37, 223 62 Lund, Sweden; 3 Department Bird Migration, Swiss Ornithological Institute, Seerose 1, 6204 Sempach, Switzerland; 4 Department of Parasitology, Faculty of Veterinary Medicine, Erciyes University, 38280 Kayseri, Turkey; 5 Vectors and Vector-Borne Diseases Implementation and Research Center, Erciyes University, 38280 Kayseri, Turkey; 6 Institute of Biodiversity and Ecosystem Research, Bulgarian Academy of Sciences, 2 Gagarin Street, 1113 Sofia, Bulgaria

**Keywords:** Avian malaria, *Haemoproteus*, *Leucocytozoon*, *Plasmodium*, Transmission areas

## Abstract

Stable isotope analysis provides valuable insights into the ecology of long-distance migratory birds during periods spent away from a specific study site. In a previous study, Swedish great reed warblers (*Acrocephalus arundinaceus*) infected with haemosporidian parasites differed in feather isotope ratios compared to non-infected birds, suggesting that infected and non-infected birds spent the non-breeding season in different locations or habitats. Here, we use a novel dataset comprising geolocator data, isotopes, and haemosporidian infection status of 92 individuals from four Eurasian populations to investigate whether parasite transmission varies with geography or habitats. We found that the probability of harbouring *Plasmodium* and *Leucocytozoon* parasites was higher in birds moulting in the eastern region of the non-breeding grounds. However, no geographic pattern occurred for *Haemoproteus* infections or overall infection status. In contrast to the previous study, we did not find any relationship between feather isotope ratios and overall haemosporidian infection for the entire current dataset. *Plasmodium*-infected birds had lower feather *δ*^15^N values indicating that they occupied more mesic habitats. *Leucocytozoon*-infected birds had higher feather *δ*^34^S values suggesting more coastal sites or wetlands with anoxic sulphate reduction. As the composition and prevalence of haemosporidian parasites differed between the old and the current dataset, we suggest that the differences might be a consequence of temporal dynamics of haemosporidian parasites. Our results emphasize the importance of replicating studies conducted on a single population over a restricted time period, as the patterns can become more complex for data from wider geographical areas and different time periods.

**Supplementary Information:**

The online version contains supplementary material available at 10.1007/s00442-023-05491-x.

## Introduction

Investigating the ecology of long-distance migratory species is challenging, because it is rarely possible to follow birds year-round. This is particularly true for songbirds that typically show weak migratory connectivity (Webster et al. 2002; Finch et al. 2017), i.e., individuals from a given breeding location commonly disperse to multiple non-breeding areas where they co-occur with birds of many different breeding origins. In the late twentieth century, researchers started to use methods for analysing the composition of stable isotopes from metabolically inert materials, such as feathers and claws (Chamberlain et al. 1996; Hobson and Wassenaar 1996). This enabled studying migratory connectivity and investigating questions which were previously difficult to address with traditional methods like the analysis of ring re-encounters. Early examples of the use of stable isotope analyses included non-breeding habitat dependent spring arrival in American redstarts *Setophaga ruticilla* (Marra et al. 1998), identification of migratory divides in willow warblers *Phylloscopus trochilus* (Chamberlain et al. 2000) and migration-dependent assortative mating in Eurasian blackcaps *Sylvia atricapilla* (Bearhop et al. 2005).

Although stable isotope analyses have provided many new and valuable insights into the annual cycle of migratory species, the method has often yielded relatively low geographic resolution even when combining the information of isotope ratios for multiple elements (Hobson et al. 2012; García-Pérez and Hobson 2014; Veen et al. 2014). Also, it is not always clear whether differences in isotope composition actually arose from tissues grown in distinct geographical areas, or from differential habitat use within a common non-breeding area (Chamberlain et al. 2000; Yohannes et al. 2008b). Such conflicting interpretations could potentially be resolved by combining isotopic data with geolocator-derived positions from the same individuals (Hallworth et al. 2013; Cherel et al. 2016; Glew et al. 2018; Seifert et al. 2018).

Identifying the non-breeding whereabouts of migratory birds is crucial not only to understand their non-breeding ecology but also for unravelling patterns of interaction between these birds and their parasites. In a previous study, haemosporidian-infected great reed warblers breeding in Sweden were found to have significantly higher feather *δ*^13^C and *δ*^15^N, and lower *δ*^2^H and *δ*^34^S values compared to non-infected birds (Yohannes et al. 2008b). Because great reed warblers undergo a complete moult in Africa (Pearson 1975; Hedenström et al. 1993), and the blood parasites infecting great reed warblers are mainly or exclusively transmitted in sub-Saharan Africa (Bensch et al. 2007), these results suggested that either the geographic location or the habitat where the birds moulted was associated with different rates of parasite transmission. An initial alternative explanation that parasite infections could directly affect the isotope values was rejected by a controlled infection experiment in moulting Eurasian siskins *Spinus spinus* (Yohannes et al. 2011), which found that even intense malaria infections did not alter feather *δ*^13^C and *δ*^15^N isotopic signatures. Finally, both stable isotope analyses (Yohannes et al. 2008a) and repeated geolocator tracking (Hasselquist et al. 2017) have revealed that great reed warblers seem faithful to their non-breeding areas in successive years. This is important, because primary haemosporidian infections typically become chronic (Asghar et al. 2015) and, therefore, infections of older birds may have originated from any of the previous non-breeding periods. Hence, a recorded infection in a particular year should still reflect the same non-breeding location as inferred from stable isotopes or geolocators in the years following the primary infection.

In the present study, we aimed at revisiting the previously observed patterns of different feather isotopic values of infected and non-infected great reed warblers (Yohannes et al. 2008b). To this end, we combined published light-level geolocation data from 92 great reed warblers from four Eurasian (Sweden, Czech Republic, Bulgaria, and Turkey) breeding populations (Koleček et al. 2016; Brlík et al. 2020) with analyses of haemosporidian infections and feather stable isotope compositions (*δ*^13^C, *δ*^15^N and *δ*^34^S) of the same individuals. The first non-breeding residency areas (moulting sites) of birds tracked from these populations are located between 5 and 15°N and span ~ 4000 km from Liberia in the west to Sudan in the east. Since the non-breeding sites are strongly overlapping for the birds from the different breeding populations (low migratory connectivity), this data set offers a novel opportunity to more deeply dissect the patterns reported by Yohannes et al. (2008b) where no information on the location of the moulting sites was available.

Based on the findings of Yohannes et al. (2008b), we predict that infected birds should have higher *δ*^13^C and *δ*^15^N values (suggesting C_4_ plant dominated and drier habitats) and lower *δ*^34^S values (suggesting more inland sites) compared with non-infected birds. If this pattern is driven by geographical variation in parasite transmission rate, we expect haemosporidian infections to be related to latitude and/or longitude of the moulting area inferred from the geolocator data. Alternatively, if parasite transmission rates differ between habitats within a common non-breeding area, we do not expect a significant relationship between the geographic position of non-breeding locations and parasite prevalence. The extent to which we will be able to disentangle the relationships between non-breeding sites and haemosporidian infections also depends on whether there are clear geographic gradients in the isotopic signatures, or whether the stable isotope ratios are more related to habitats within the geographic regions.

## Materials and methods

We used data pertaining to 92 adult great reed warblers (36 females, 55 males, and 1 unsexed) equipped with light-level geolocators in 2008–2016 at four breeding sites: Sweden (SE, Lake Kvismaren; 59°10ʹ N, 15°24ʹ E; *n* = 35), Czech Republic (CZ, Hodonínské and Mutěnické ponds; 48°53ʹ N, 17°03ʹ E; *n* = 34), Bulgaria (BG, Kalimok wetlands; 44°00ʹ N, 26°26ʹ E; *n* = 19), and Turkey (TR, Cernek Gölü, Kızılırmak Deltası, 41°39ʹ N, 36°02ʹ E; *n* = 4). The birds were captured using mist nets, sexed based on the shape of the cloacal protuberance and the presence of brood patch, and aged according to Svensson (1992). The geolocators were retrieved in the following year, with the exception of four birds that were recaptured after 2 years. For detailed numbers of deployed and retrieved devices, see ESM Table S1. Basic technical information on the devices is specified in Koleček et al. (2016, 2018), Brlík et al. (2020) and Emmenegger et al. (2021).

Along with the light data for geolocating their non-breeding grounds, there were also blood and feather samples available for all these 92 birds. Blood was sampled from the brachial vein both before deployment and after retrieval of the geolocators, and stored in SET buffer or absolute EtOH for molecular analysis of haemosporidian parasites. Upon geolocator retrieval, we also sampled a third tail feather (SE and TR), a second tertial (CZ), or the distal part of a fifth primary (BG) for stable isotopic analysis. All these feathers are assumed to be grown in Africa during the first part of the non-breeding period when the great reed warblers conduct their complete feather moult (De Roo and Deheegher 1969; Pearson 1975; Hanmer 1979; Bensch et al. 1991; Hedenström et al. 1993). We also collected each of the three feather types from 30 adult individuals breeding in the SE, CZ, and BG populations in 2018 to check for intra-individual variation in stable isotope signatures. No statistically significant effect of feather type on stable isotope composition was observed using a series of three simple linear mixed-effects models for each stable isotope (*δ*^13^C, *δ*^15^N, *δ*^34^S) with feather type as a fixed effect (factor with three levels) and individual identity as a random intercept (feather type; *δ*^13^C:* F*_2,58_ = 0.26, *P* = 0.772; *δ*^15^N: *F*_2,58_ = 1.39, *P* = 0.256; *δ*^34^S: *F*_2,58_ = 3.05, *P* = 0.055; ESM Fig. S1).

We determined the spatiotemporal migration patterns using GeoLight 1.03 (Lisovski and Hahn 2012), following the procedure given in Emmenegger et al. (2014). In short, we applied the threshold method to determine sunrise and sunset times from the geolocator-recorded light data for each day (Hill 1994). Then, each geolocator was calibrated, by calculating an individual sun elevation angle (SEA) from the light data recorded during the post-breeding and (if available) pre-breeding period (in-habitat calibration; Lisovski et al. 2012). The resulting SEAs varied between − 6.5° and 3.1° depending upon the type of geolocator, habitat, and individual bird behaviour. After excluding sun events outside two interquartile ranges (*k*) with the loessFilter function, we used the SEAs to determine stationary periods using the changeLight function (threshold = 0.9 quantile of change point probability, minimum stationary period = 3 days). We merged stationary periods when average positions of consecutive non-breeding sites were not farther than approximately 200 km. We used an average of the individual SEAs obtained from on-bird calibration for calculating sub-Saharan non-breeding locations. We defined the position of each non-breeding site as the peak of the frequency distributions (mode) of both latitudes and longitudes of the daily positions within this stationary period. In this study, we used the location of the first non-breeding site as the measure of the geographic position of a bird’s wintering site. The first non-breeding site is used by the great reed warblers during their complete feather moult that is conducted from mid-October to mid-December (Jenni and Winkler 2020). We chose to use primarily the location of the first non-breeding site, because this is the period when the feathers used in the isotope analyses were growing and the birds are stationary for several months (median 89 days, IQR 34 days, min 57 days, max 238 days) allowing for rather precise geolocator-based estimates of longitude and latitude. For additional details on geolocator specifications and return rates, see Koleček et al. (2016, 2018) and Brlík et al. (2020).

To assess the haemosporidian infection status of the sampled birds, DNA was extracted and purified using standard protocols described previously (Yohannes et al. 2008b; Ciloglu et al. 2019) and diluted to a concentration of 25 ng/µl. We employed a multiplex PCR protocol (Ciloglu et al. 2019) to screen the samples for genus-specific infections of *Haemoproteus*, *Plasmodium*, and *Leucocytozoon* parasites. This protocol has been shown to be highly effective at detecting and identifying both single and mixed infections from all three haemosporidian genera (Ciloglu et al. 2019). All samples were also analysed by standard nested PCR (Hellgren et al. 2004) followed by sequencing of positive samples with the forward primer using Big-Dye on an ABI PRISM™ 3100 sequencer (Applied Biosystems, FL, USA). Finally, the derived chromatograms were edited in Geneious v. R11 (https://www.geneious.com) and the sequences compared against parasite lineages registered in the MalAvi database (Bensch et al. 2009).

To determine the known transmission areas of the parasite lineages detected, we also queried the MalAvi database (Bensch et al. 2009), which can be searched for parasite lineages found in obligate resident species or in juveniles of migratory species to delimit potential transmission areas. For each parasite lineage, we collated records of locally hatched juveniles and/or adults of all obligate resident species, as detection of parasites in these individuals indicates local transmission. While we acknowledge the recent taxonomic revisions that have placed certain *Haemoproteus* lineages into the genus *Parahaemoproteus* (Galen et al. 2018), we have chosen to retain the designation *Haemoproteus* to maintain consistency with the nomenclature used in Yohannes et al. (2008b). This facilitates direct comparisons of our findings with previous research. Any references to *Haemoproteus* in our work should be interpreted in this context.

Prior to stable isotope analysis, feathers were washed in 2:1 chloroform:methanol solution for 24 h, then rinsed with distilled water, and left to air-dry for 24 h. Feather keratin samples of about 0.3 mg, pre-weighed in tin cups, were combusted using the vario micro-cube elemental analyser (Elementar, Analysensysteme, Germany) and the resultant CO_2_, N_2_, and SO_2_ gases were introduced into a Micromass Isoprime isotope ratio mass spectrometer (Isoprime, Cheadle Hulme, UK) via a continuous flow-through inlet system. Sample ^13^C/^12^C, ^15^N/^14^ N, and ^34^S/^32^S ratios are expressed in the conventional delta (*δ*^13^C, *δ*^15^N, and *δ*^34^S) notation in parts per million (‰). These values are relative to the following standards: the Vienna Pee Dee Belemnite (VPDB) for carbon, atmospheric N_2_ for nitrogen, and sulphanilamide-calibrated and traceable to NBS-127 (barium sulphate, *δ*^34^S =  + 20.3‰) for sulphur. Internal laboratory standards indicate that our measurement errors (SD) were ± 0.15‰, 0.05‰, and 0.05‰ for *δ*^15^N, *δ*^13^C, and *δ*^34^S, respectively. Stable isotope analysis was conducted in the Stable Isotope Laboratory at the Institute of Limnology, University of Konstanz, Germany.

For all subsequent analyses, we summarised the parasite infection status across both sampling occasions (geolocator deployment and retrieval). When the bird was scored as infected at least once, we treat the bird as infected (if the bird was scored as infected in the first year, but as non-infected in the second year, we assume that the infection was not detected in the second year). To test for the effect of geographic position of the moulting site and habitat used during feather growth, we fitted a binomial generalised linear model in the brms package (Bürkner 2021; for details, see below) with overall blood parasite infection status (0—uninfected, 1—infected) as a binary response variable and longitude and latitude of the first non-breeding site as well as stable isotope ratios (*δ*^13^C, *δ*^15^N, and *δ*^34^S) from feathers moulted in Africa as predictors. To directly compare the current results with the results of Yohannes et al. (2008b; where they did not screen for *Leucocytozoon* infections and had only one sampling occasion), we additionally fitted a separate model for the Swedish birds wherein we did not consider the detection of *Leucocytozoon* infections and used only the infection status (by genera *Haemoproteus* and/or *Plasmodium*) upon geolocator deployment.

We also fitted an analogical multilevel (multiple-response) model with genus-specific infection status (0—uninfected, 1—infected) for each genus as the response variable and the same predictors. The models were formulated in the brms package (Bürkner 2021). This package enables flexible model specification and model estimates are conducted in Stan using Markov chain Monte Carlo (MCMC) sampling via adaptive Hamiltonian Monte Carlo (Hoffman and Gelman 2014; Stan Development Team 2021). The binary responses were specified as Bernoulli (0/1) response and we used default non-informative priors. Models were run with a total of 2 chains for 2000 iterations each, with a burn-in period of 1000 iterations per chain, which was sufficient to achieve adequate mixing and convergence (all $\widehat{R}$ values were equal to 1.00; for trace plots see ESM Fig. S2). Predictors were considered statistically significant if the 95% credible intervals did not include zero. To test whether isotopic signatures reflect geography, we used Pearson’s correlation between feather *δ*^13^C, *δ*^15^N, and *δ*^34^S values with latitude and longitude of the first non-breeding site. All data analyses were conducted in R (R Core Team 2020).

## Results

**Fig. 1:**
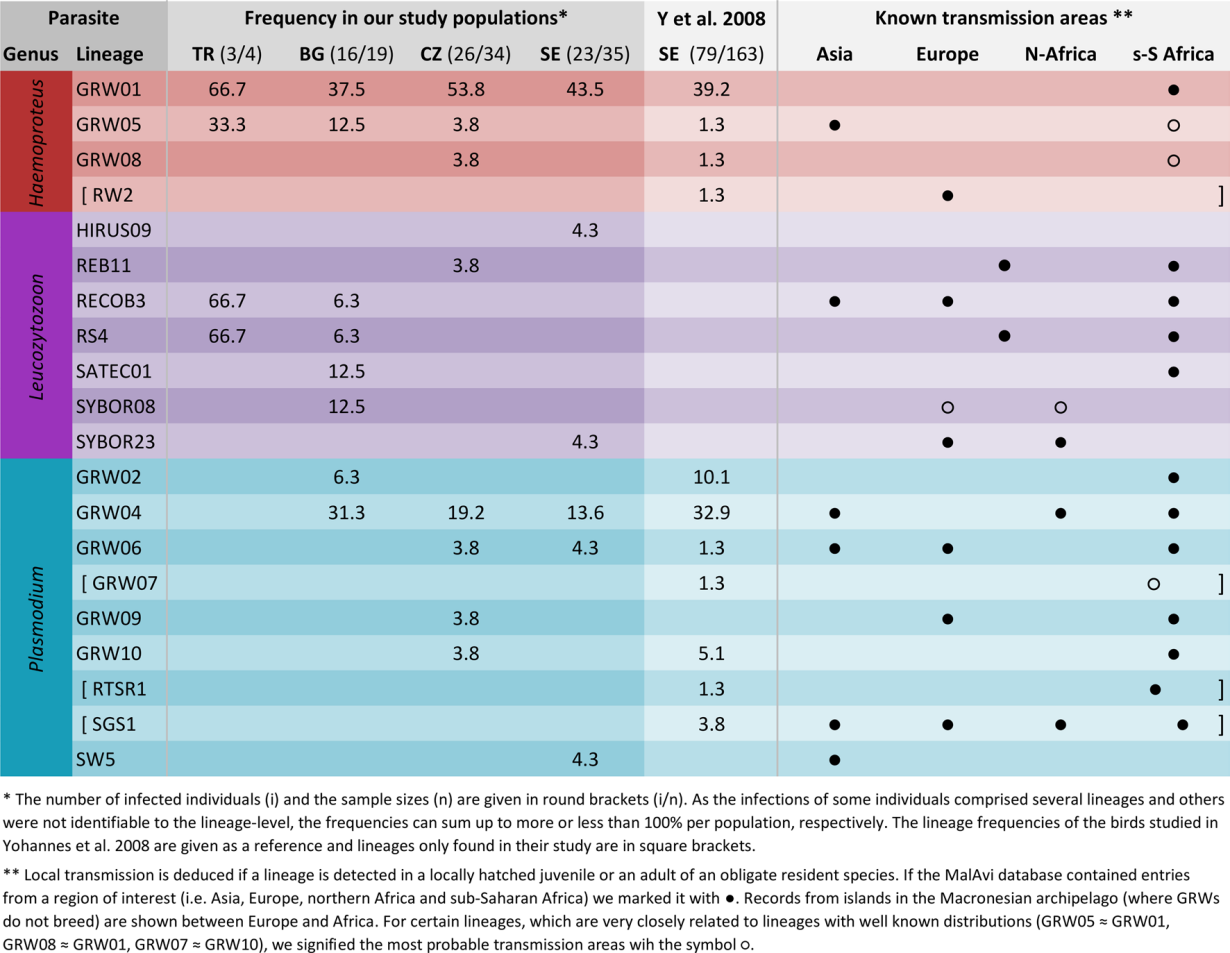
Overview of haemosporidian lineages and the frequencies with which we detected them among the infected individuals in our study. TR—Turkey, BG—Bulgaria, CZ—Czech Republic, and SE—Sweden. For a reference, we also list the lineage frequencies as determined by Yohannes et al. (2008b) and the known transmission areas derived by records of these lineages in the MalAvi database (Bensch et al. 2009)

Overall, 74% of the 92 individuals were scored positive for blood parasite infection. The prevalence did not significantly differ between the sampling sites (SE: 66%, CZ: 76%, BG: 84%, TR: 75%; Fisher’s exact test *P* = 0.499). The infections comprised 36 *Haemoproteus*, 45 *Plasmodium*, 9 *Leucocytozoon*, and 19 mixed-genus infections (13 *Haemoproteus* and *Plasmodium*, 2 *Plasmodium* and *Leucocytozoon*, 1 *Haemoproteus* and *Leucocytozoon*, and 3 infected with all three genera). The query of the MalAvi database revealed that most of the common parasite lineages have known transmission areas in sub-Saharan Africa. Only parasite lineages rare to great reed warbler have well-documented transmission areas outside the non-breeding range of great reed warblers. For an overview of all the parasite lineages and their known transmission areas, see Fig. 1.

**Fig. 2:**
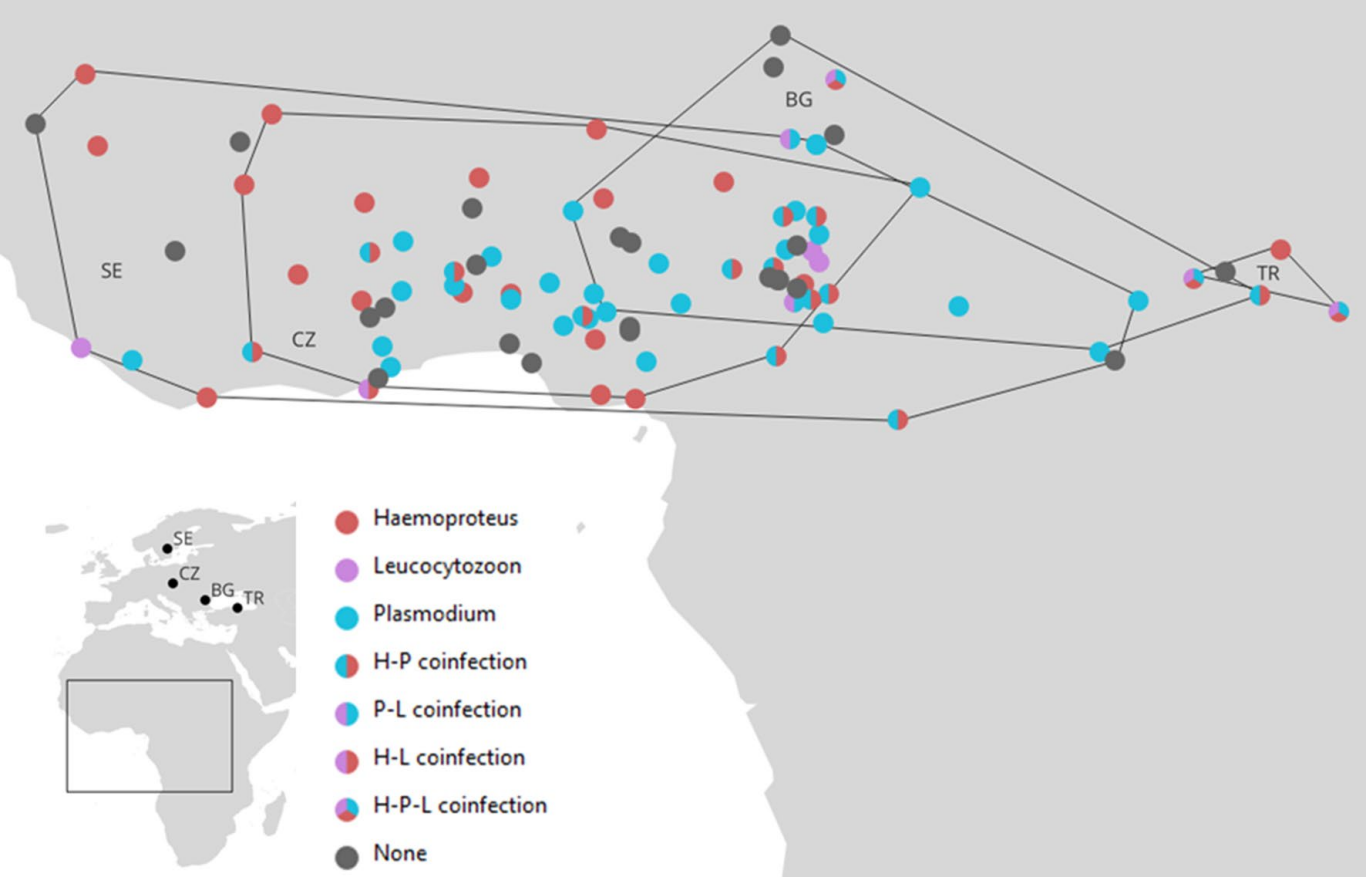
Location of first sub-Saharan non-breeding grounds of great reed warblers from four breeding populations (Sweden—SE, Czech Republic—CZ, Bulgaria—BG, and Turkey—TR) infected by different haemosporidian parasites of the genera *Haemoproteus* (H), *Plasmodium* (P), and *Leucocytozoon* (L). None—non-infected individuals

The first non-breeding sites (i.e., moulting sites) spanned across an extensive part of sub-Saharan Africa and showed varying degrees of overlap between the breeding populations (Fig. 2). Feather *δ*^13^C values did not statistically significantly correlate with the location of the first non-breeding site (latitude: *r* = 0.026, *P* = 0.809; longitude: *r* = 0.139, *P* = 0.185). Feather *δ*^15^N values weakly positively correlated with latitude (*r* = 0.235, *P* = 0.024) but not with longitude (*r* = 0.002, *P* = 0.986). Feather *δ*^34^S values did not significantly correlate with latitude (*r* = − 0.148, *P* = 0.162) but were negatively related to longitude of the first non-breeding site (*r* = − 0.46, *P* < 0.001).

There was no significant effect of the first non-breeding site location and feather stable isotope profiles on overall blood parasite infection (Table 1a). At the genus-specific level, *Plasmodium-*infected birds were located more to the east during the non-breeding period than birds without *Plasmodium* infections ($\widehat{\beta }$ = 0.08 ± 0.03, 95% CrI: 0.02, 0.15; Table 1b, Fig. 2, ESM Fig. S3). Similarly, *Leucocytozoon-*infected birds tended to be located more to the east during the non-breeding period than birds without *Leucocytozoon* infections ($\widehat{\beta }$ = 0.10 ± 0.05, 95% CrI: 0, 0.21; Table 1b). Moreover, *Plasmodium-*infected birds had lower feather *δ*^15^N values than birds without *Plasmodium* infections ($\widehat{\beta }$ = − 0.27 ± 0.15, 95% CrI: − 0.58, − 0.01; Table 1b) and *Leucocytozoon-*infected birds had higher feather *δ*^34^S values than birds without *Leucocytozoon* infections ($\widehat{\beta }$ = 0.65 ± 0.35, 95% CrI: 0.02, 1.39; Table 1b). There were no statistically significant differences between birds infected and non-infected by *Haemoproteus* in the location of non-breeding sites or feather isotope profiles (Table 1b). There was no statistically significant effect of the second non-breeding site location on overall blood parasite infection or genus-specific infection status (Table S2).

**Table 1:** Results of binomial GLMs with longitude and latitude of the first non-breeding sites, as well as feather *δ*^13^C, *δ*^15^N, and *δ*^34^S values explaining the variation in (a) overall haemosporidian infection, (b) genus-specific infection status (multiple-response GLM: Haem—*Haemoproteus*, Plas—*Plasmodium*, Leuc—*Leucocytozoon*), and (c) overall haemosporidian infection status (*Haemoproteus* and *Plasmodium* only—see the section “Materials and methods”) in the Swedish subsample for comparative purposes with the previous study by Yohannes et al. (2008b)

	$\widehat{\beta }$ ± SE	LL	UL
(*a*)* Overall infection status*			
Intercept	3.37 ± 3.02	− 2.35	9.31
Latitude	− 0.05 ± 0.09	− 0.22	0.12
Longitude	0.02 ± 0.03	− 0.04	0.09
δ^13^C	0.14 ± 0.08	− 0.02	0.29
δ^15^N	− 0.16 ± 0.15	− 0.46	0.12
δ^34^S	0.23 ± 0.20	− 0.16	0.62
(*b*)* Genus-specific infection status*			
Intercept (Haem)	− 2.05 ± 2.58	− 7.06	3.11
Intercept (Plas)	2.02 ± 2.76	− 3.20	7.42
Intercept (Leuc)	− 11.51 ± 5.41	− 22.98	− 1.27
Latitude (Haem)	− 0.01 ± 0.08	− 0.18	0.14
Longitude (Haem)	− 0.01 ± 0.03	− 0.06	0.05
δ^13^C (Haem)	0.03 ± 0.07	− 0.11	0.16
δ^15^N (Haem)	0.12 ± 0.14	− 0.13	0.40
δ^34^S (Haem)	0.12 ± 0.17	− 0.21	0.44
Latitude (Plas)	− 0.06 ± 0.08	− 0.23	0.10
**Longitude (Plas)**	**0.08 ± 0.03**	**0.02**	**0.15**
δ^13^C (Plas)	0.02 ± 0.07	− 0.12	0.15
**δ**^**15**^**N (Plas)**	− **0.27 ± 0.15**	− **0.58**	− **0.01**
δ^34^S (Plas)	0.11 ± 0.18	− 0.21	0.48
Latitude (Leuc)	0.04 ± 0.15	− 0.27	0.31
**Longitude (Leuc)**	**0.10 ± 0.05**	**0.00**	**0.21**
δ^13^C (Leuc)	− 0.02 ± 0.11	− 0.26	0.21
δ^15^N (Leuc)	0.17 ± 0.25	− 0.33	0.63
**δ**^**34**^**S (Leuc)**	**0.65 ± 0.35**	**0.02**	**1.39**
(*c*)* Overall infection status Sweden*			
Intercept	6.16 ± 5.86	− 4.99	18.22
Latitude	0.10 ± 0.16	− 0.22	0.42
**Longitude**	**0.14 ± 0.08**	**0.01**	**0.31**
δ^13^C	0.17 ± 0.16	− 0.12	0.50
**δ**^**15**^**N**	− **1.02 ± 0.39**	− **1.89**	− **0.32**
δ^34^S	0.50 ± 0.49	− 0.39	1.56

$\widehat{\beta }$ denotes the posterior mean, and LL and UL represent lower and upper limits of the 95% credible interval, respectively. Statistically significant values are shown in bold

In terms of the overall infection status of Swedish birds upon geolocator deployment (excluding *Leucocytozoon* infections to make the results comparable to Yohannes et al. 2008b), infected birds had lower feather *δ*^15^N values ($\widehat{\beta }$ = − 1.02 ± 0.39, 95% CrI: − 1.89, − 0.32) and tended to be located more to the east than uninfected birds ($\widehat{\beta }$ = 0.14 ± 0.08, 95% CrI: 0.01, 0.31; Table 1c).

## Discussion

In the present study, we revealed several associations between haemosporidian infections, geography, and habitat use during the non-breeding period by combining data from light-level geolocation and stable isotope analysis. The well-known migratory system of the great reed warbler and its diverse haemosporidian parasites allowed not only to take a more nuanced view on genus-specific associations between infections and habitat use, but also to compare the Swedish part of the newly acquired data (collected 2008–2016) with a study conducted earlier on the same population (1999–2004; Yohannes et al. 2008b). Importantly, the current dataset provides geographic information on the location of non-breeding sites of individual birds screened for haemosporidian infections that was not available for birds examined by Yohannes et al. (2008b). Interestingly, the findings in the current study do not support the relationships between haemosporidian infection status and stable isotope values found by Yohannes et al. (2008b).

### Associations between infections, non-breeding geography, and habitat use

The population-specific overall prevalence did not significantly differ, even though these birds originate from distant parts of the species’ breeding range, indicating that most of these parasites are transmitted on the tropical non-breeding grounds, where individuals from all study populations partly overlap. Earlier investigations conducted in the Sahel region were able to determine transmission areas of some of the haemosporidian lineages commonly found to infect great reed warblers (e.g., the *Haemoproteus* lineage GRW1 and the *Plasmodium* lineage GRW4) by sampling resident bird species in Africa (Waldenström et al. 2002). This was further supported by our query of the MalAvi database (Bensch et al. 2009) that revealed that most of the haemosporidian lineages we found have known transmission areas in sub-Saharan Africa and only few of the rather low-prevalent parasite lineages have documented transmission outside the non-breeding areas (Fig. 1). This pattern corroborates that the few low-prevalence parasite lineages transmitted outside the non-breeding period should not severely affect the main results of this study and that non-breeding locations and habitats are good candidates for factors influencing the risk of infection in these migratory hosts.

Although the overall prevalence was similar, the prevalence of the three haemosporidian genera as well as the relative frequencies of the parasite lineages differed substantially among populations. These differences can be partly attributed to the parallel migration pattern of the great reed warbler (Koleček et al. 2016) but are, at the same time, likely obscured by the largely overlapping non-breeding ranges of adjacent breeding populations (Fig. 2). Nevertheless, neither the geographic location nor the isotopic signature of the non-breeding site was related to the overall blood parasite infection status. Even though birds from individual breeding populations spread across large parts of sub-Saharan Africa (Fig. 2), the local conditions at these sites might differ less than expected by the mere geographic distance of individual non-breeding sites, as great reed warblers tend to favour wetlands and tall grasslands year-round (Dyrcz 2020). Alternatively, the resolution of light-level geolocation and stable isotope analysis may not be fine enough to capture the general associations between the risk of infection by haemosporidian parasites and the isotopic origin of diet, as well as the actual habitat patches used during the non-breeding season (see also the section “Limitations and conclusions”). Also, if a considerable proportion of the infections were transmitted elsewhere than at the moulting sites of the feathers that were analysed for stable isotopes, this could explain the lack of correlation between infection status and isotopic signature (see also the section “Limitations and conclusions”).

However, when looking at the level of parasite genera, we found several correlations between feather isotopic signatures and parasite infection status providing coherent explanations for the different habitat requirements of the vector groups that transmit the different parasite genera at the African non-breeding sites. *Plasmodium*-infected birds had significantly lower feather *δ*^15^N values. This indicates that, compared to birds without *Plasmodium* infections, they occupied more mesic habitats (Heaton et al. 1986; Sealy et al. 1987; van der Merwe et al. 1990; Ambrose 1991). This linkage might be mostly driven by insect vectors, as *Plasmodium* parasites are mainly transmitted by mosquitoes of the family Culicidae. Compared to Ceratopogonidae and Hippoboscidae (typical vectors of *Haemoproteus* parasites) and Simuliidae (major vectors of *Leucocytozoon* parasites), mosquitoes are closely associated with natural or artificial water bodies as habitat for their larvae (Laird 1988; Gu et al. 2006). Our finding is thus in line with the results of a meta-analysis demonstrating that the global distributions of different haemosporidian genera are shaped by different climatic and environmental variables. The meta-analysis showed that the distribution of *Plasmodium* parasites, but not of other parasite genera, was governed primarily by wetland availability and vegetation density (Fecchio et al. 2021).

In contrast, *Leucocytozoon*-infected birds had higher feather *δ*^34^S values, indicating that they moult closer to the coast where the *δ*^34^S values are known to be enriched in ^34^S compared to inland areas (Lott et al. 2003; Zazzo et al. 2011; Brlík et al. 2022), or in wetlands where sulphate reduction may occur under largely anaerobic conditions (Thode 1991). Nevertheless, these conditions do not fully correspond to the most common environmental requirements of simuliid flies, the vectors of *Leucocytozoon*, whose larvae are adapted to lotic waters and are typically confined to mountain and foothill streams, although some species occur at large rivers and can tolerate poor water quality (Palmer and de Moor 1998). It is possible that certain *Leucocytozoon* vectors in sub-Saharan Africa have more diverse habitat preferences than currently understood, or that other environmental or ecological factors play a role in shaping these patterns. Future investigations are needed to fully understand the complex relationships between *Leucocytozoon* parasites, their vectors and the moulting habitats of their bird hosts. In the above mentioned global meta-analysis, *Leucocytozoon* distribution was mostly driven by elevation and rain (Fecchio et al. 2021).

We also found that the probability of harbouring *Plasmodium* was higher in birds moulting in the eastern part of the non-breeding grounds and a similar tendency was detected for *Leucocytozoon*-infected birds. In contrast, there was neither such a geographic pattern for *Haemoproteus*-infected individuals nor for the overall infection status of the birds examined. Even though the migratory connectivity in great reed warblers is known to be rather weak, there is still a parallel migration pattern with the birds roughly maintaining the longitudinal arrangement of their breeding populations at their stopovers and non-breeding sites (Koleček et al. 2016). Therefore, some of the relationships between infections and non-breeding longitude could also be related to the conditions at the distinctly separated breeding sites. However, because most of the common parasite lineages are thought to be solely transmitted in sub-Saharan Africa, the latter explanation can only be relevant for some less-well-known parasite lineages, particularly for those which seem to have well-established transmission areas outside sub-Saharan Africa (see Fig. 1). For future haemosporidian studies, we urge for more focus on screening also individuals of local (e.g., African) resident species, rather than solely wintering individuals of (e.g., Palaearctic) migrant species, to get a clearer picture of the transmission areas of the growing number of known haemosporidian lineages.

### Comparison of the Swedish population with the data from Yohannes et al. (2008b)

When comparing the infections of the Swedish great reed warblers sampled in this study with those in Yohannes et al. (2008b) collected about 15 years earlier, there is a notable difference both in overall infection prevalence and parasite assemblage (Fig. 1). In particular, the *Plasmodium* lineages GRW2 and GRW4 made up a lower proportion among the infected birds in the recent Swedish data set (0% and 13.6%) compared to the data set of Yohannes et al. 2008b (10.1% and 32.9%). This indicates that a large proportion of the variation is probably due to temporal changes in the host–parasite–vector system. A relevant comparison can only be made for *Haemoproteus* and *Plasmodium* [routinely detected by a nested PCR protocol described in Hellgren et al. (2004)], as Yohannes et al. (2008b) did not screen for *Leucocytozoon* parasites (detected in the current study by a newer multiplex PCR protocol; Ciloglu et al. 2019). While Yohannes et al. (2008b) detected infections in 48% of the screened individuals, the Swedish breeding birds in the present study had an overall prevalence of 66% of which 63% were infected by at least one of the two parasite genera that Yohannes et al. (2008b) also screened for. Although the detection method used in the current study (multiplex PCR; Ciloglu et al. 2019) has a slightly higher sensitivity than the nested PCR used by Yohannes et al. (2008b), this did not lead to a statistically significant difference in overall prevalence determined by the two methods in the samples comparatively analysed by Ciloglu et al. (2019). Our current double-sampling design revealed 13 instances where birds, having initially tested positive, later tested negative. These instances may hint at false negatives. Although we cannot directly estimate the proportion of false negatives from a single sample of the previous study by Yohannes et al. (2008b), this may add to the explanation of the discrepancy in prevalence between the two studies. The single sampling by Yohannes et al. (2008b) might have missed certain chronic infections, not detectable in the blood or where infection intensity was so low that the single nested PCR reaction failed to detect it. Therefore, contrasting studies with different methodologies necessitates caution. Moreover, the difference in prevalence that varied from 48 to > 60% is largely in line with the findings of a past study on the temporal dynamics of haemosporidian infections in the same Swedish great reed warbler breeding population, which found prevalence to be fluctuating and slightly increasing over time (Bensch et al. 2007).

However, in terms of changes in the parasite assemblage, methodological differences could contribute more substantially to the differences between Yohannes et al. (2008b) and the present study. Considering the large proportion of co-infections with several parasite genera in the current study, the improved separation of co-infections achieved by the multiplex PCR approach could lead to a different relative frequency of both parasite genera and lineages. While the nested PCRs detect *Haemoproteus* and/or *Plasmodium* together in one reaction, there is a risk that the reactions favour one of the two genera in a mixed-genus infection (often the infection with the higher infection intensity). However, as 8 of the 11 parasite lineages found by Yohannes et al. (2008b) were not detected in the current Swedish dataset, the effect of the two detection protocols is likely small (note that the higher number of parasite lineages detected by Yohannes et al. 2008b can be mainly due to lower sample size in the current Swedish data). Overall, the differences between the previous and the current data rather suggest that this host–parasite system is quite dynamic and may show changes in prevalence of individual haemosporidian lineages within timeframes of about 2 decades.

The spatiotemporal dynamics in drought and rain periods in sub-Sahara Africa (Berntell et al. 2018; Ekolu et al. 2022) could also explain some of the variation between Yohannes et al. (2008b) and the current study. Furthermore, the current study used only individuals that survived and successfully returned with geolocators, which could potentially have led to a sample biased towards more resilient or successful individuals compared to Yohannes et al. (2008b), which was not part of a tracking study. It is also interesting to note that GRW2 is known to have negative consequences for the great reed warbler host (Westerdahl et al. 2005; Asghar et al. 2011, 2012), which may result in a lower return rate to the next breeding season, thus possibly explaining the very low prevalence of this *Plasmodium* lineage in the current data set.

### Limitations and conclusions

Our study provided the first insights into the parasite genus-specific relationships between infection patterns and isotopic signatures in the feathers of the hosts. Besides these specific findings, we also discuss some limitations of our study system that may have led to differences between the current study and the study by Yohannes et al. (2008b) and why we could not provide more definitive answers to the question of how geography or non-breeding habitat relates to blood parasite infections.

It has been shown that great reed warblers are not stationary during the entire period they spent south of the Sahara but often undertake intra-tropical movements (Lemke et al. 2013; Hasselquist et al. 2017; Koleček et al. 2018). After crossing the Sahara in August/September, they typically stay at a first non-breeding site in the Sahel until November or December, where they most likely undergo complete moult (De Roo and Deheegher 1969; Pearson 1975; Bensch et al. 1991; Hedenström et al. 1993). It cannot be fully excluded, however, that some individuals may have arrested moult and finished it at the second non-breeding ground that can be 250–2500 km away from the first non-breeding site (Koleček et al. 2018) and thus, in such cases, the feather isotope profiles would not match the first non-breeding site. This is, however, not likely for the Swedish birds, because in 40 years of studying this population, no individuals have been observed with two ‘types’ of flight and tail feathers that differ in shading and wear (own unpublished observations). Similarly, some of the infections might not stem from the first non-breeding site but from any subsequent site which could introduce noise to our data and weaken any associations between geography, habitat, and haemosporidian infection. However, note that the location of the second non-breeding sites did not appear to predict haemosporidian infections (ESM Table S2).

We also acknowledge that the positioning precision of light geolocation is limited (Lisovski et al. 2012) and we may therefore be unable to geographically match possible vector habitats, such as patchy wetlands within an otherwise dry area. The currently available remedy for this limitation would be to use miniature archival GPS devices that allow much higher spatial resolution (Hallworth and Marra 2015; Yanco et al. 2022). Finally, feather stable isotope signatures may reflect the local habitat more or less accurately depending upon the mobility of flying insects between isotopically different habitats (Quinby et al. 2020).

Even if our study could not fully disentangle the causes of the parasite genus-specific patterns we found, it provides a first indication that habitat use within moulting sites might contribute to the infection patterns found in great reed warblers. There are also hints towards parasite genus-specific relationships probably related to vector-specific habitat requirements. In the context of the comparison of the current study with the previous study by Yohannes et al. (2008b), we argue that upscaling studies from a single population and a few years is important as the resulting patterns can be more complex for data from wider geographical areas and longer time periods. Future tagging with small programmable archival GPS tags could yield precise locations of transmission areas, allowing for a more accurate appraisal of differences between parasitized and unparasitized individuals. In addition, future studies focusing on co-infections would be particularly interesting as they could shed light on potential interactions between different parasite lineages, providing a more comprehensive understanding of the dynamics and impact of multiple haemosporidian infections on their avian hosts.

### Supplementary Information

Below is the link to the electronic supplementary material.

Supplementary file 1 (DOCX 403 KB)

## Data Availability

The datasets used during the current study are available from the corresponding author on reasonable request.

## Code availability

The R code is available from the corresponding author on reasonable request.
