# Supplementary material for: The association between haemosporidian infection and non-breeding moult location in great reed warblers revisited by combining feather stable isotope profiles and geolocator data
Source: Oecologia. 2023 Dec 23;204(1):107–18. doi: 10.1007/s00442-023-05491-x (PMC10830769; doi:10.1007/s00442-023-05491-x)
Supplement: Supplementary file 1 — (DOCX 403 KB) [file 442_2023_5491_MOESM1_ESM.docx]

**Electronic Supplemental Material**

The association between haemosporidian infection and non-breeding moult location in great reed warblers revisited by combining feather stable isotope profiles and geolocator data

Petr Procházka*, Tamara Emmenegger, Silke Bauer, Arif Ciloglu, Dimitar Dimitrov, Bengt Hansson, Dennis Hasselquist, Elizabeth Yohannes, Pavel Zehtindjiev, Staffan Bensch

* Correspondence: prochazka@ivb.cz

**Table S1** Numbers of deployed tags, returned tagged birds and retrieved geolocators at each study site, and the numbers of individuals used in the analysis. Note that only birds with complete data on haemosporidian infection, stable isotope composition, and non-breeding site positions were included in the current study. M – males, F – females, U - unsexed

| Site | Deployed in | No. deployed | Birds returned | Tags retrieved | No. used | Reference |
| --- | --- | --- | --- | --- | --- | --- |
| Sweden | 2008 | 9 M, 0 F | 2 M, 0 F | 2 M, 0 F | 1 | Lemke et al. (2013) |
|  | 2009 | 26 M, 0 F | 8 M, 0F | 8 M, 0 F | 2 | Lemke et al. (2013) |
|  | 2010 | 22 M, 20 F | 10 M, 5 F | 10 M, 5 F | 13 | unpubl. |
|  | 2011 | 16 M, 0 F | 4 M, 0 F | 4 M, 0 F | 3 | Koleček et al. (2016) |
|  | 2012 | 23 M, 0 F | 8 M, 0 F | 8 M, 0 F | 3 | Koleček et al. (2016) |
|  | 2013 | 16 M, 14 F | 7 M, 3 F | 7 M, 3 F | 6 | unpubl. |
|  | 2014 | 10 M, 10 F | 1 M, 5 F | 1 M, 5 F | 4 | unpubl. |
|  | 2015 | 2 M, 12 F | 0 M, 4 F | 0 M, 4 F | 2 | unpubl. |
|  | 2016 | 0 M, 6 F | 0 M, 1 F | 0 M, 1 F | 1 | unpubl. |
| Czechia | 2012 | 16 M, 16 F | 8 M, 4 F | 4 M, 4 F | 6 | Požgayová et al. (2022) |
|  | 2013 | 33 M, 38 F | 12 M, 14 F | 0 M, 0 F* | 0 | Požgayová et al. (2022) |
|  | 2014 | 46 M, 24 F | 13 M, 8 F | 10 M, 6 F | 9 | Požgayová et al. (2022) |
|  | 2015 | 34 M, 16 F | 10 M, 5 F | 10 M, 5 F | 13 | Požgayová et al. (2022) |
|  | 2016 | 29 M, 11 F | 5 M, 1 F | 4 M, 1 F | 6 | Požgayová et al. (2022) |
| Bulgaria | 2012 | 22 M, 10 F | 4 M, 1 F | 3 M, 1 F | 0 | Koleček et al. (2016) |
|  | 2015 | 51 M, 19 F | 7 M, 7 F | 7 M, 7 F | 12 | Emmenegger et al. (2021) |
|  | 2016 | 43 M, 32 F, 5 U | 8 M, 2 F | 8 M, 2 F | 7 | Emmenegger et al. (2021) |
| Turkey | 2013 | 12 M, 0 F | 4 M, 0 F | 4 M, 0 F | 4 | Koleček et al. (2016) |
|  |  |  |  |  |  |  |

* All the 26 birds returned without devices due to problems with harness material

**Table S2** Results of binomial GLMs with longitude and latitude of *second* non-breeding sites explaining the variation in (a) overall haemosporidian infection, and (b) genus-specific infection status (multiple-response GLM: Haem – *Haemoproteus*, Plas – Plasmodium, Leuc – *Leucocytozoon*).
$\hat{\beta}$ denotes the posterior mean, LL and UL represent lower and upper limits of the 95% credible interval, respectively

|  | $\hat{\beta}$ ± SE | LL | UL |
| --- | --- | --- | --- |
| *a) Overall infection status* |  |  |  |
| Intercept | 1.48 ± 0.75 | 0.11 | 2.98 |
| Latitude | –0.08 ± 0.09 | –0.26 | 0.08 |
| Longitude | –0.00 ± 0.03 | –0.06 | 0.05 |
|  |  |  |  |
| *b) Genus-specific infection status* |  |  |  |
| Intercept (Haem) | 0.81 ± 0.68 | –0.48 | 2.17 |
| Intercept (Plas) | –0.10 ± 0.63 | –1.28 | 1.13 |
| Intercept (Leuc) | –3.58 ± 1.39 | –6.47 | –1.07 |
| Latitude (Haem) | –0.15 ± 0.08 | –0.32 | 0.01 |
| Longitude (Haem) | –0.03 ± 0.03 | –0.08 | 0.02 |
| Latitude (Plas) | –0.03 ± 0.08 | –0.18 | 0.11 |
| Longitude (Plas) | 0.03 ± 0.03 | –0.02 | 0.08 |
| Latitude (Leuc) | –0.13 ± 0.12 | –0.36 | 0.10 |
| Longitude (Leuc) | 0.10 ± 0.06 | –0.01 | 0.21 |

**Fig. S1.** Boxplots depicting stable isotope values (*δ*^13^C, *δ*^15^N, *δ*^34^S) of the three different feather types sampled in 30 great reed warbler individuals (see Materials and methods). Thick lines show medians, boxes interquartile ranges (IQR), whiskers the most extreme values within 1.5 IQR, and dots outliers

**Fig. S2.** Density and trace plots of Bayesian multilevel GLMs fitted using the brms package, with longitude and latitude of the first non-breeding sites, as well as feather *δ*^13^C, *δ*^15^N, *δ*^34^S values explaining the variation in (a) overall infection status, (b) genus-specific infection status (multiple response binomial GLM: Haem – *Haemoproteus*, Plas – *Plasmodium*, Leuc – *Leucocytozoon*), and (c) overall haemosporidian infection status (*Haemoproteus* and *Plasmodium* only – see Methods) in the Swedish subsample for comparative purposes with the previous study by Yohannes et al. (2008b). Each panel shows the density and trace plot for a specific model parameter.

a) Overall infection status

b) Genus-specific infection status

(c) Overall haemosporidian infection status (*Haemoproteus* and *Plasmodium* only) in the Swedish subsample

**Fig. S3.** Non-breeding sites of great reed warblers from four breeding populations in Sweden (SE, green), Czech Republic (CZ, blue) Bulgaria (BG, pink) and Turkey (TR, red). Closed (infected) and open (uninfected) symbols indicate total infection status (top left) and infection with the genera *Haemoproteus* (top right), *Plasmodium* (bottom left) and *Leucocytozoon* (bottom right)


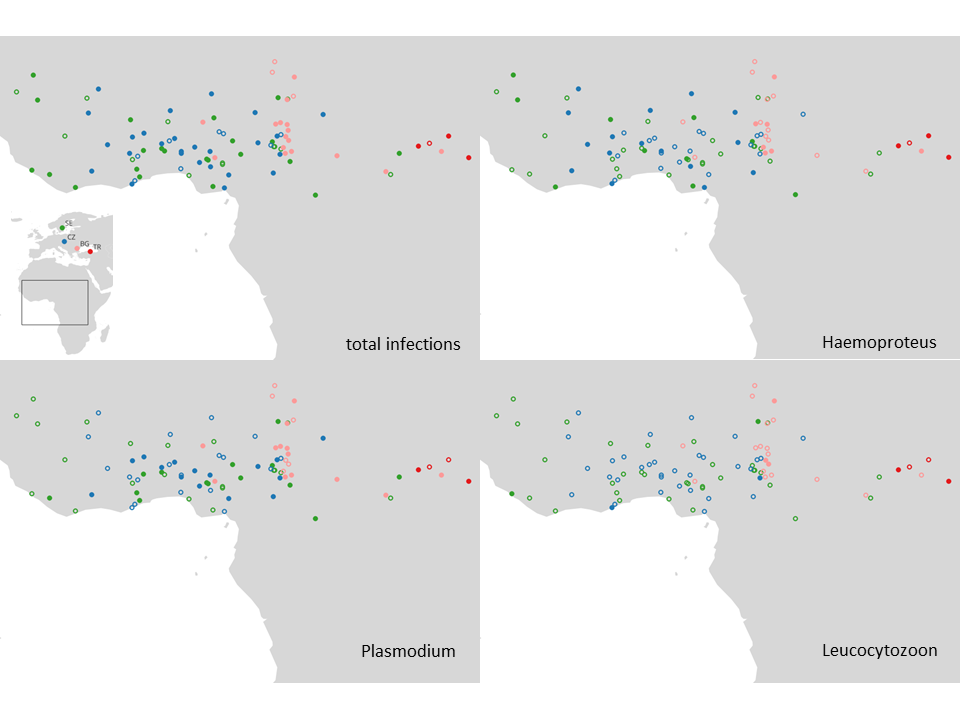


**References**

Emmenegger T, Bensch S, Hahn S, Kishkinev D, Procházka P, Zehtindjiev P, Bauer S (2021) Effects of blood parasite infections on spatiotemporal migration patterns and activity budgets in a long‐distance migratory passerine. Ecol Evol 11:753–762 doi: 10.1002/ece3.7030

Koleček J, Procházka P, El‐Arabany N, Tarka M, Ilieva M, Hahn S, Honza M, de la Puente J, Bermejo A, Gürsoy A, Bensch S, Zehtindjiev P, Hasselquist D, Hansson B (2016) Cross‐continental migratory connectivity and spatiotemporal migratory patterns in the great reed warbler. J Avian Biol 47:756–767 doi: 10.1111/jav.00929

Lemke HW, Tarka M, Klaassen RH, Åkesson M, Bensch S, Hasselquist D, Hansson B (2013) Annual cycle and migration strategies of a trans-Saharan migratory songbird: a geolocator study in the great reed warbler. PLoS One 8:e79209 doi: 10.1371/journal.pone.0079209

Požgayová M, Koleček J, Honza M, Procházka P (2022) Is there an effect of fostering a brood parasite on the timing of host autumn migration? J Ornithol 163:417–423 doi: 10.1007/s10336-021-01949-y
